# Aging Impairs Reverse Remodeling and Recovery of Ventricular Function after Isoproterenol-Induced Cardiomyopathy

**DOI:** 10.3390/ijms23010174

**Published:** 2021-12-24

**Authors:** Laia Yáñez-Bisbe, Anna Garcia-Elias, Marta Tajes, Isaac Almendros, Antonio Rodríguez-Sinovas, Javier Inserte, Marisol Ruiz-Meana, Ramón Farré, Núria Farré, Begoña Benito

**Affiliations:** 1 Cardiovascular Research Group, Vascular Biology and Metabolism, Vall d’Hebron Research Institute (VHIR), 08035 Barcelona, Spain; laiayb19@gmail.com (L.Y.-B.); antonio.rodriguez.sinovas@vhir.org (A.R.-S.); Javier.inserte@vhir.org (J.I.); mruizmeana@gmail.com (M.R.-M.); 2 Laboratory of Molecular Physiology, Department of Experimental and Health Sciences, Universitat Pompeu Fabra, 08003 Barcelona, Spain; anna80@gmail.com; 3 Bio-Heart Cardiovascular Diseases Research Group, Bellvitge Biomedical Research Institute (IDIBELL), L’Hospitalet de Llobregat, 08908 Barcelona, Spain; cicelyflyn@gmail.com; 4 Unit of Biophysics and Bioengineering, School of Medicine and Health Sciences, University of Barcelona, 08036 Barcelona, Spain; isaac.almendros@ub.edu (I.A.); rfarre@ub.edu (R.F.); 5 Centro de Investigación Biomédica en Red de Enfermedades Respiratorias (CIBER-ES), 28029 Madrid, Spain; 6 Institute for Biomedical Research August Pi i Sunyer (IDIBAPS), 08036 Barcelona, Spain; 7 Centro de Investigación Biomédica en Red de Enfermedades Cardiovasculares (CIBER-CV), Instituto de Salud Carlos III, 28029 Madrid, Spain; 8 Department of Cardiology, Hospital del Mar, 08003 Barcelona, Spain; 9 Heart Diseases Biomedical Research Group (GREC), IMIM (Hospital del Mar Medical Research Institute), 08003 Barcelona, Spain; 10 Department of Medicine, Universitat Autònoma de Barcelona, 08023 Barcelona, Spain; 11 Cardiology Department, Hospital Vall d’Hebron, 08035 Barcelona, Spain

**Keywords:** heart failure, HFrEF, ageing, cardiac remodeling, ventricular dysfunction, reverse remodeling, female animals

## Abstract

Information about heart failure with reduced ejection fraction (HFrEF) in women and the potential effects of aging in the female heart is scarce. We investigated the vulnerability to develop HFrEF in female elderly mice compared to young animals, as well as potential differences in reverse remodeling. First, HF was induced by isoproterenol infusion (30 mg/kg/day, 28 days) in young (10-week-old) and elderly (22-month-old) female mice. In a second set of animals, mice underwent isoproterenol infusion followed by no treatment during 28 additional days. Cardiac remodeling was assessed by echocardiography, histology and gene expression of collagen-I and collagen-III. Following isoproterenol infusion, elderly mice developed similar HFrEF features compared to young animals, except for greater cell hypertrophy and tissue fibrosis. After beta-adrenergic withdrawal, young female mice experienced complete reversal of the HFrEF phenotype. Conversely, reversed remodeling was impaired in elderly animals, with no significant recovery of LV ejection fraction, cardiomyocyte hypertrophy and collagen deposition. In conclusion, chronic isoproterenol infusion is a valid HF model for elderly and young female mice and induces a similar HF phenotype in both. Elderly animals, unlike young, show impaired reverse remodeling, with persistent tissue fibrosis and cardiac dysfunction even after beta-adrenergic withdrawal.

## 1. Introduction

Heart failure (HF) is a major public health problem associated with high morbidity and mortality, particularly in the aging population [1]. The interplay between HF and aging happens at several levels. First, HF prevalence increases with age, with 2% of affected individuals in the general population and up to >8% of those aged ≥75 years [2,3]. Aging also worsens HF prognosis, having been independently associated with increased readmission and mortality rates [3]. Furthermore, aging could also play a role in the response to HF therapy, as suggested by recent studies showing that, in patients with HF and reduced ejection fraction (HFrEF), younger age is associated with greater improvement of ventricular function after pharmacological or device therapy or after left ventricular assist device (LVAD) support [4,5]. Several factors have been advocated to explain the different behavior of HF according to age, such as the coexistence of comorbidities, the loss of functional exercise capacity and, more importantly, the presence of aging-associated vascular, cellular and interstitial molecular changes at the cardiac level that result in stiffer and less compliant hearts with greater vulnerability to functional decline [6,7].

Despite previous information supporting the existence of age-related differences in HFrEF, most clinical studies have been performed in relatively young populations, leaving little evidence-based data to establish recommendations for HF management in the elderly [8,9]. Investigating the role that aging plays in the development and recovery of HF in clinical studies is limited by the presence of comorbidities and other individual or group factors, potentially leading to bias. Animal models could provide further insights [10], but, as for the clinical setting, most experimental research in HF has been performed using relatively young animals [11]. Therefore, current experimental data on HF does not have a direct translation into all clinical scenarios of human HF.

Gender also has a role in the HF phenotype. Women are characteristically more prone to develop HF with preserved EF (HFpEF) [11] and, as a result, have been consistently underrepresented in clinical HF trials of HFrEF, where most of the classical HF evidence stands [9]. Among patients with HFrEF, sex differences have been described concerning HFrEF pathophysiology, presentation, and morbidity and mortality [12,13]. Overall, women with HFrEF are older and have more comorbidities but present better response to therapy and survival, compared to men [12,13]. However, as in the clinical setting, most experimental research in HF has been performed in male animals. In this context, the need to extend the research on HFrEF to women and female animals has been claimed in recent years [14,15].

Previous studies suggest that the cardiac response to a noxa may considerably vary depending on age, indicating that cardiovascular remodeling might be conditioned by the age at which exposure to the primary stimulus occurs [16]. Furthermore, data from clinical studies suggest that aging could also have an impact on cardiac recovery of ventricular dysfunction [4,5]. However, no definite information in this regard is available for women. Accordingly, using exclusively female mice, we sought to analyze whether exposure to chronic beta-stimulation, a well characterized HF model, induces HF to a similar extent in young and elderly animals. The second and main aim of the study was to evaluate in female mice the role of aging on cardiac reverse remodeling and HF recovery after releasing the primary HF-inducing stimulus.

## 2. Results

### 2.1. Isoproterenol Infusion Induces HF with Subtle Particularities in Elderly Female Mice

**Figure 1:**
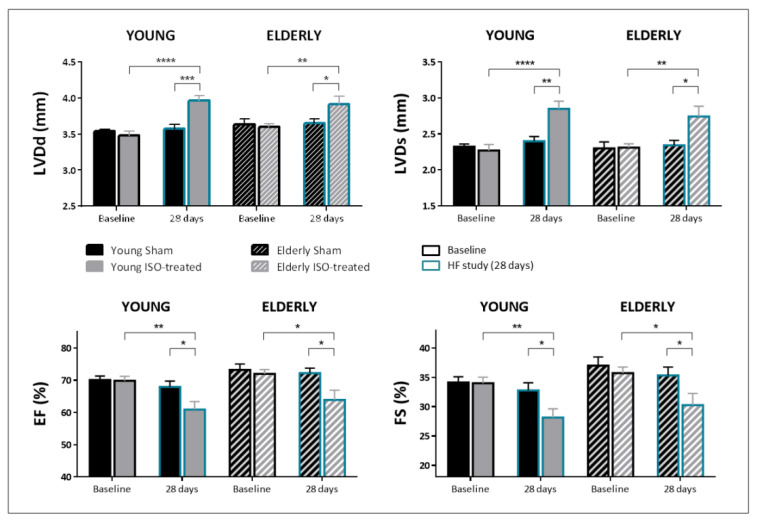
Echocardiographic parameters at baseline (black outlined bars) and after 28-day exposure to saline or isoproterenol infusion (blue outlined bars) in young and elderly mice. LVDd: End-diastolic left ventricular diameter; LVDs: End-systolic left ventricular diameter; IVS: interventricular septum thickness; PW: posterior wall thickness; EF: ejection fraction; FS: fractional shortening. N = 13 in both groups of young mice, and N = 14 in both groups of elderly mice. * *p* < 0.05, ** *p* < 0.01, *** *p* < 0.001, **** *p* < 0.0001.

Figure 1 shows the echocardiographic results of the first set of animals, designed to assess the cardiac effects induced by isoproterenol infusion in young and elderly female mice. No differences were observed at baseline among the four study groups (Figure 1, black-outlined bars). As expected in the young population, isoproterenol infusion induced a considerable increase in both diastolic and systolic LV dimensions and also marked reductions in ejection fraction and fractional shortening, changes that were not observed in the sham group (Figure 1, solid bars). Isoproterenol induced similar morphologic changes in the elderly mice (Figure 1, stripped bars), with a non-significant trend to develop less ventricular dilation compared to the young (9.1% change of LVDd in elderly vs. 14.1% change in young animals, p NS). The decrease of ejection fraction was virtually of the same magnitude in young and elderly mice (−11.8% vs. −11.2% respectively).

**Figure 2:**
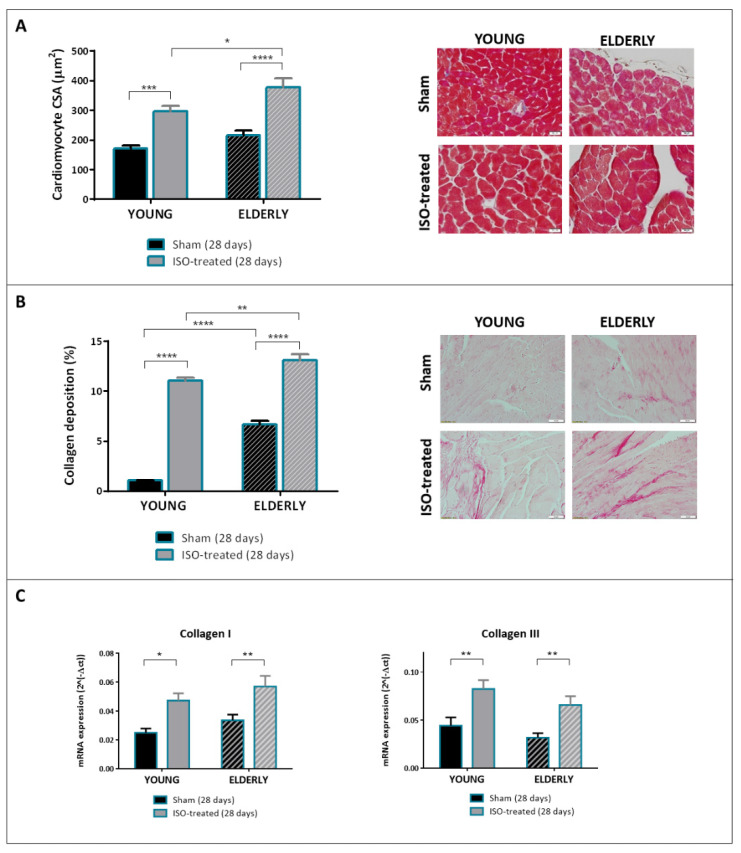
Histological findings and mRNA expression analyses at 28 days after exposure to isoproterenol or saline infusion in young and elderly mice. (**A**) Cardiomyocyte cross-sectional area (CSA) in the four study groups. A representative microphotograph for each one of them is shown (magnification ×40). N = 7/5 in sham/ISO-treated young mice; N = 7/5 in sham/ISO-treated elderly mice. (**B**) Quantification of collagen deposition in picrosirius-stained sections in the four study groups. Representative microphotographs are shown (magnification ×20). N = 5/7 in sham/ISO-treated young mice; N = 7/5 in sham/ISO-treated elderly mice. (**C**) mRNA expression of collagen I and collagen III in the four study groups. N = 8/6 in sham/ISO-treated young mice; N = 8/6 in sham/ISO-treated elderly mice. * *p* < 0.05, ** *p* < 0.01, *** *p* < 0.001, **** *p* < 0.0001.

Histological and gene expression analyses at 28 days are shown in Figure 2. As expected, young mice under isoproterenol infusion (solid bars) showed cardiomyocyte hypertrophy (Figure 2A) and histological fibrosis (Figure 2B) compared to controls. So did elderly mice of the ISO-treated group (stripped bars), although both cardiomyocyte CSA (376 μm^2^ vs. 296 μm^2^) and collagen deposition (13.1% vs. 11.1%) were significantly higher in elderly versus young mice receiving isoproterenol (Figure 2A,B, gray bars). It is important to note, however, that elderly animals in the sham group had significantly greater cardiac fibrosis compared to young controls (6.7% vs. 1.1%, respectively, Figure 2, black bars). Therefore, the magnitude of increase of tissue fibrosis in mice subjected to isoproterenol versus controls appeared greater in young than in elderly mice (by ~10 fold in young compared to a ~2 fold in elderly mice). As shown in Figure 2C, mRNA expression of collagen I and collagen III was increased in both ISO-treated groups compared to controls, with no relevant differences according to age.

Together, these results support the suitability of the isoproterenol model as a HF model in elderly female mice. Despite exhibiting greater ventricular fibrosis at baseline, elderly female animals developed HF features in response to isoproterenol, including LV dilation and dysfunction and increased histological fibrosis. Elderly mice under isoproterenol also exhibited certain subtle particularities in terms of LV remodeling, including the presence of greater cell hypertrophy and tissue fibrosis compared to young mice.

### 2.2. Reverse Remodeling Is Distinctly Different in Young and Elderly Female Mice

**Figure 3:**
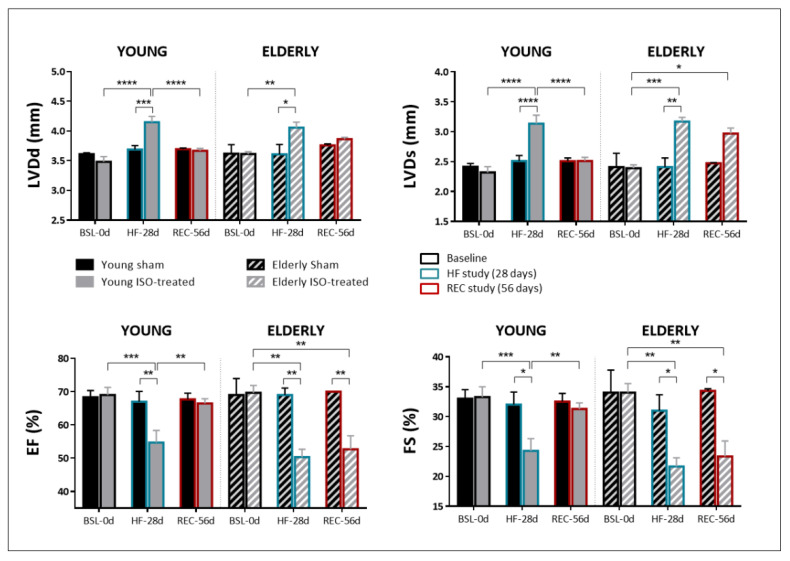
Echocardiographic parameters at baseline (black outlined bars), after 28-day exposure to saline or isoproterenol infusion (HF-28d, blue outlined bars) and at 56 days after exposure + recovery period (REC-56d, red outlined bars) in young and elderly mice. LVDd: End-diastolic left ventricular diameter; LVDs: End-systolic left ventricular diameter; IVS: interventricular septum thickness; PW: posterior wall thickness; EF: ejection fraction; FS: fractional shortening). N = 6/7 in sham/ISO-treated young mice; N = 3/4 in sham/ISO-treated elderly mice. * *p* < 0.05, ** *p* < 0.01, *** *p* < 0.001, **** *p* < 0.0001.

The study of reverse remodeling and HF recovery was assessed in a second set of female animals in whom isoproterenol infusion was withdrawn after 28 days of exposure. Figure 3 summarizes the results of the echocardiographic parameters obtained in the four study groups at the three study timepoints: baseline (BSL-0d), end of isoproterenol/sham challenge (28 days, HF induction, HF-28d) and end of recovery period (56 days, recovery, REC-56d). Young animals with isoproterenol exposure and subsequent withdrawal exhibited complete reverse remodeling, with LVDd and LVDs values at REC-56d close to those at BSL-0d (Figure 3, solid gray bars). Conversely, LVDd and particularly LVDs failed to return to baseline values in elderly mice after isoproterenol withdrawal (Figure 3, stripped gray bars). Furthermore, EF and FS recovered in young animals (values of 66.4% ± 3.8 and 31.3% ± 2.7 at REC-56d, respectively) but failed to do so in the elderly (values of 52.7% ± 7.0 and 23.3% ± 4.5, at REC-56d, respectively).

**Figure 4:**
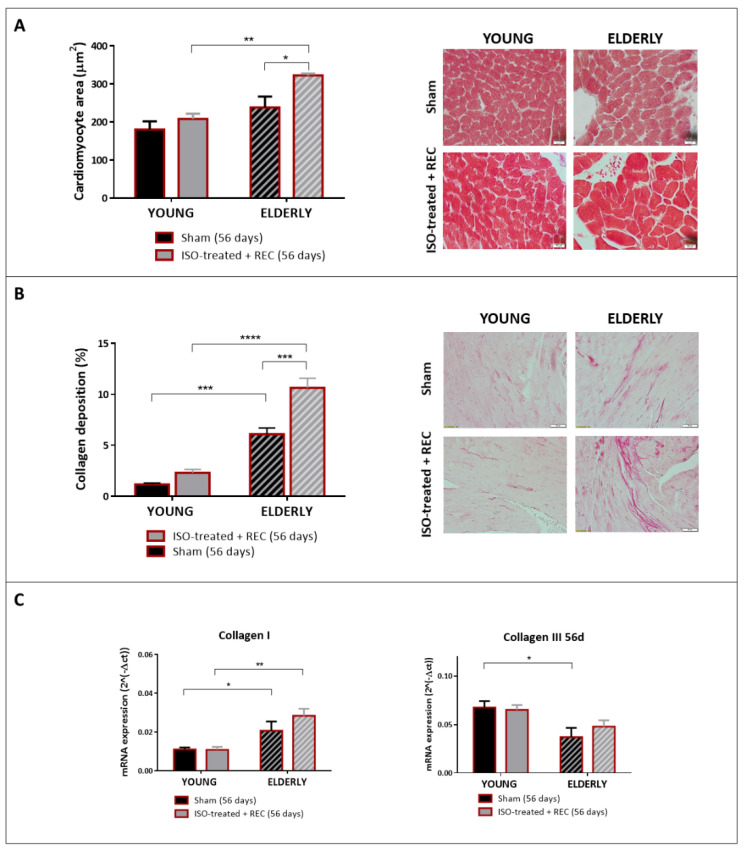
Histological findings and mRNA expression analyses at 56 days, after exposure to isoproterenol or saline infusion and recovery, in young and elderly mice. (**A**) Cardiomyocyte cross-sectional area (CSA) in the four study groups. A representative microphotograph for each one of them is shown (magnification ×40). N = 5/5 in sham/ISO-treated young mice; N = 3/4 in sham/ISO-treated elderly mice. (**B**) Quantification of collagen deposition in picrosirius-stained sections in the four study groups. Representative microphotographs are shown (magnification ×20). (**C**) mRNA expression of collagen I and collagen III in the four study groups. N = 6/7 in sham/ISO-treated young mice; N = 3/4 in sham/ISO-treated elderly mice. * *p* < 0.05, ** *p* < 0.01, *** *p* < 0.001, **** *p* < 0.0001.

Histological analyses of LV tissue sections at 56 days showed that young female animals undergoing isoproterenol infusion and subsequent recovery had similar cardiomyocyte CSA and collagen deposition compared to their counterpart sham (Figure 4A,B, solid bars). Conversely, elderly female mice (stripped bars) showed cardiomyocyte hypertrophy and significantly greater collagen deposition compared to their corresponding sham and also compared to young mice undergoing the same protocol (isoproterenol infusion and recovery period). Collagen I expression was found increased in elderly mice compared to young ones, particularly in those having been treated with isoproterenol, and collagen III was downregulated in old versus young sham mice (Figure 4C).

These results highlight the existence of distinct particularities regarding reverse remodeling according to age in female animals: whereas young mice can fully recover from HF after releasing the primary stimulus, reverse remodeling seems to be markedly impaired in elderly female animals regarding both morphological and functional parameters. This distinct behavior is accompanied by increased collagen I expression in elderly mice.

## 3. Discussion

The results of our study indicate that chronic beta-stimulation, a well characterized HF model in young male animals, induces a HF remodeling both in young and elderly female mice, with the latter displaying slightly distinct features such as greater cell hypertrophy and tissue fibrosis. More importantly, our work suggests that aging markedly determines HF reverse remodeling after releasing the primary stimulus: whereas young female animals exhibited full recovery of functional and structural parameters, both at the macroscopic and the cellular level, elderly mice showed persistent cell hypertrophy, tissue fibrosis and cardiac dysfunction upon beta-adrenergic withdrawal.

HF is a major public health problem affecting almost 40 million individuals worldwide [1]. Although the relevance of HFpEF is increasingly recognized, HFrEF remains the most common HF form reported in contemporary registries [17] and is associated with higher mortality in follow-up [18]. An advanced age and female sex have been associated with greater risk of HFpEF, and on the other hand, both have been consistently underrepresented in clinical trials of HFrEF [11,12]. Similarly, previous experimental research in HF has been majorly performed on young male animals [11]. However, recent real-life data indicate that, among HFrEF patients, 21.9% are aged >75 and 21.6% are women [17], which still represent significant numbers of affected individuals given the major burden of HF in the community.

We therefore carried out the present work to assess the particularities of HF induction and recovery at different lifespan time-points using exclusively female mice. We used a well-established experimental model of HF that closely recapitulates the clinical features of human chronic HF [19]. At baseline, young and elderly female mice had no perceptible differences in echocardiographic parameters, but histological analyses showed a trend to higher cardiomyocyte CSA and significantly higher collagen deposition in elderly versus young animals. A previous work performed in mice which aimed to evaluate the changes in physiological cardiac remodeling throughout life showed that both cardiomyocyte hypertrophy and fibrosis increase beyond 18 months of age [13]. These findings are consistent with the morphological changes seen in the aging human heart, which contribute to cardiac functional deterioration and HF development under chronic hemodynamic overload [6].

In our study, female young mice developed a typical HF phenotype in response to chronic isoproterenol infusion, a finding that has also been reported in males and is consistent with some available prior publications [19,20,21]. However, whether the extent of morphological changes would be comparable between males and females undergoing isoproterenol infusion remains to be established. Although some studies have reported similar features between male and female animals receiving isoproterenol for HF induction [21], a recent study using transgenic mice suggested that the HF phenotype was enhanced in females compared to males [22], in association with a greater activation of the renin-angiotensin system. More importantly, HF traits, including LV dilation and dysfunction, cardiomyocyte hypertrophy and cardiac fibrosis, were also seen in elderly female mice in response to isoproterenol. Along with fibrosis, gene expression of collagen I and III, indicative of fibroblast activation, was increased in elderly animals receiving isoproterenol. These findings are novel since most experimental models of HF to date have been developed in young animals [23]. The few data coming from studies with male rats ageing 18 months at most suggest that both young and elderly animals would have similar response to beta-adrenergic stimulation [24]. However, no previous information exists comparing the HFrEF progression in young and aged (22 months old) female mice under isoproterenol treatment. Our results therefore validate the isoproterenol HF model in elderly animals, even though it has been reported that aging is associated with beta-adrenergic receptor dysfunction, expression and desensitization [25], particularly in women [26]. Our results indicate that, despite an age-related remodeling of beta-adrenergic receptors, chronic beta-adrenergic stimulation still leads to similar structural and functional changes in old and young animals.

Very notably, our study confirmed a different behavior between young and elderly mice after removal of beta-adrenergic stimulation. Whereas young female mice experienced complete recovery of heart dimensions and functionality, elderly animals showed persistent left ventricular dilation and dysfunction, cardiomyocyte hypertrophy and tissue fibrosis. These findings were accompanied by increased expression of collagen I, with no relevant changes in collagen III. Aging has been associated with changes in the relative proportion of collagen I and III within the myocardium [27], and specifically HF has been associated with predominant expression of collagen I [28]. Collagen I fibers exhibit relatively higher stiffness, whereas collagen III fibers have higher susceptibility to plastic deformation [29]. Although the mechanisms by which collagen I, but not collagen III, persisted elevated in elderly mice were not addressed, a relative increase in expression of collagen I over collagen III would render the myocardium less distensible in these animals and is in agreement with previous data [28].

Importantly, although some previous studies have evaluated the effects of reverse remodeling following different therapies for HF, to the best of our knowledge no previous experimental study has evaluated the potential differences in HF recovery according to age following withdrawal of the primary stimulus. Because we only used female animals, whether the differences that were observed between young and elderly animals were exclusively due to age or other factors (such as hormonal influence) remains to be established. Studying the effects of age in HF recovery in male animals and/or ovariectomized females could bring out some answers in this regard.

Cardiac reverse remodeling, understood as the restoration of chamber geometry and, at the cellular level, decrease of cell size and tissue fibrosis in previously failing hearts, has not been thoroughly studied. The available data majorly come from clinical studies on response to medical or cardiac resynchronization therapy (CRT) [5,30]. However, two settings such as LVAD implant (with the consequent mechanical cardiac unloading) and ablation of premature ventricular complexes (PVC) in PVC-induced tachycardiomyopathy represent better clinical examples where reverse remodeling could occur after removal of the primary HF-inducing stimulus rather than in response to therapy. Clinical reports have suggested that both LVAD implant and PVC ablation are associated with recovery of LV dysfunction [5,30,31,32], and, at least for post-LVAD remodeling, partial regression of tissue fibrosis and gene expression [5,33], findings that are consistent with those observed in our young animals.

Importantly, the clinical setting has also highlighted that myocardial reverse remodeling might not always occur to the same extent in all individuals. A non-ischemic etiology and a shorter HF duration are known factors associated with better response to pharmacological or device therapy [4,5]. Despite their underrepresentation in HFrEF trials, women seem to exhibit similar reverse remodeling than men after medical therapy [12], although some small reports have pointed to a greater response to CRT [34]. Finally, there exists little information on the characteristics of reverse remodeling according to age. Several studies suggest that elderly patients might respond comparably to the young after pharmacological therapy or CRT [35,36,37]. Conversely, data coming from large registries of LVAD support that the incidence of cardiac recovery is 2-fold more likely in individuals aged <50 years [33,35]. Likewise, ablation of PVCs leads more frequently to reversal of LV dysfunction in younger individuals [38]. With the use of an animal model, we could provide new insights into the reverse remodeling in young and elderly animals and confirm that it is considerably impaired in the aging heart.

Our work has several limitations. We only used female animals. Although gender differences might exist in HF induction and recovery, our first aim was to assess differences in remodeling according to age and not to sex. We chose to use female animals because women and female animals have been underrepresented in clinical and experimental studies dealing with HFrEF, and solid information in this population is lacking. Our results, therefore, might not be necessarily extendible to cardiac remodeling in males. Although variable, it has been reported that menopausal transition might be present in 25–40% of animals in aging colonies [39]. Therefore, it is possible that menopause or some degree of hormonal fluctuations are present in some of our animals. However, our aim was to describe the events occurring in female animals throughout life concerning HF induction and recovery, and menopause is part of this process. Cardiomyocyte CSA was measured in sections stained with Masson’s Trichrome. Although a validated approach, other methods, like immunostaining with Troponin and Wheat Germ Agglutinin (WGA), might be more accurate. Only some aspects concerning cardiac remodeling were assessed, including ventricular chamber dimensions and function, cardiomyocyte hypertrophy, interstitial fibrosis and gene expression of some fibrotic markers. More specific cellular and molecular mechanisms associated with this process were not investigated. Constitutional aging is accompanied by sarcomeric and gap junction remodeling and changes in extracellular matrix composition, among others [7]. Whether these or other mechanisms participate in the compromised reverse remodeling seen in our elderly animals should be addressed in future studies.

## 4. Materials and Methods

### 4.1. Animals

The study was carried out on 10-week-old (young) and 22-month-old (elderly) C57BL/6 female mice (Charles River Laboratories, Saint Germain sur L’Arbresle, France), a strain previously validated for the study of age-dependent cardiac remodeling [13]. The study was approved by the Ethics Committee for Animal Experimentation of the University of Barcelona and the Hospital del Mar Medical Research Institute. All animals were kept with undisturbed social interaction in conventional cages under a 12-h light/dark cycle and ad libitum access to tap water and standard diet.

### 4.2. Study Design

A first series of experiments was addressed to characterize the isoproterenol HF model in elderly mice. To this end, young and elderly female animals were both randomized to receive whether chronic infusion of isoproterenol, a validated model mimicking human chronic HF (ISO-treated) [19], or saline (sham). Osmotic mini-pumps (1004, Alzet) were implanted subcutaneously under anesthesia with 1.25% isoflurane, as previously described [19]. Briefly, a small incision was made on the back of each animal, and the skin was brought apart from the underlying connective tissue with blunt-ended scissors to expose the subcutaneous tissue, where the pumps were implanted. Pumps were filled to release continuously isoproterenol (Sigma Aldrich) dissolved in 0.9% NaCl at a dose of 30 mg/kg/day over 28 days [19]. The same saline without isoproterenol was used for the sham group. At the final timepoint, animals were euthanized by an intraperitoneal injection of sodium pentobarbital (100 mg/kg), and hearts were quickly excised. The left ventricle (LV) tissue was snap-frozen in liquid nitrogen and stored at −80 °C until usage, and midventricular LV slices were fixed in 4% paraformaldehyde for histological studies.

In a second set of experiments, addressed to study the effects of aging on recovery from ISO-induced HF, the same osmotic pumps delivering 30 mg/kg/day of isoproterenol or 0.9% NaCl over 28 days were implanted in young and elderly mice. Mice were subsequently kept for 28 additional days without receiving any treatment. All animals underwent an echocardiographic study before sacrifice at day 56, and heart samples were collected as described above.

### 4.3. Echocardiography

Repeated transthoracic echocardiographic studies were performed in young and elderly mice in the two sets of experiments: the HF-induction set (baseline, and 28 days) and the HF-recovery set (baseline, 28 days and 56 days). Echocardiograms were carried out under general anesthesia with 2% isoflurane using a Vivid IQ and L8-18i-D Linear Array 5–15 MHz (General Electric Healthcare, Horten, Norway). Mice were placed in supine position on a continuously warmed platform to maintain body temperature, and the four limbs were fixed. Ultrasound gel was applied on the left hemithorax and hearts were imaged in parasternal short-axis projections. M-mode echocardiograms of the mid-ventricle were recorded at the level of the papillary muscles. The left ventricular end-diastolic (LVDd) and end-systolic (LVDs) internal diameters were measured in the M-mode recordings. LV fractional shortening (FS) was calculated by (LVDd–LVDs)/LVDd × 100%. Ejection fraction (EF) was calculated by the formula packed in GE Healthcare Ultrasound Vivid 7 system and proposed by the American Society of Echocardiography. The average of 3 consecutive cardiac cycles was used for each measurement. All measures were taken blinded.

### 4.4. Histology

Heart samples fixed in buffered 4% paraformaldehyde were embedded in paraffin and cut into 5 µm-thick slices. Heart sections were deparaffinized and rehydrated with xylene, ethanol (100, 90 and 70%) and water. LV cardiomyocyte cross-sectional area (CSA) was measured in transverse sections stained with Masson’s Trichrome Stain Kit. At least 30 random cardiomyocytes from each slice were measured at 400× magnification using the Image J software (Image J, U.S. National Institutes of Health, Bethesda, MD, USA). To assess LV fibrosis, tissue sections were stained with Picrosirius red for quantification of collagen deposition, as previously described [40]. Ten representative ventricular photomicrographs per animal were acquired at 40× with an Olympus B × 60 microscope and a QI-maging Q-cam and quantified as percentage of collagen deposition with an automated color recognition processing plugin from Image J. Perivascular, pericardial and endocardial collagen was excluded from measurements. All measures were analysed blinded to group assignment.

### 4.5. Real-Time PCR

Frozen LV tissue samples were homogenized with a mortar and a pestle in liquid nitrogen. RNA was extracted using the Nucleospin RNA kit (Macherey-Nagel, Düren, Germany), which includes a DNAse I treatment, and retrotranscribed into cDNA with the High Capacity RNA-to-cDNA kit (Thermofisher, Waltham, MA, USA). Quantitative RT-PCR was performed using the Taqman Universal PCR master mix (Thermofisher, Waltham, MA, USA) on the 7900HT Fast Real-Time PCR System (Applied Biosystems, Waltham, MA, USA). Primers used for COL1A1 (Mm0080166_g1), COL3A1 (Mm00802300_m1) and GAPDH (Mm99999915_g1) were purchased from Thermofisher. Gene expression was normalized to GAPDH expression and represented as 2^ΔCt^ [41].

### 4.6. Statistical Analyses

Data are presented as mean ± SEM. Statistical analyses were performed using two-way ANOVA: for the echocardiographic parameters, factors were timepoint and group (4 study groups according to ISO treatment and age); for histological and expression analyses, factors were treatment and age group. All analyses were followed by Bonferroni post hoc correction when interaction was found (GraphPad Prism 6.0). Differences were considered statistically significant when *p* < 0.05.

## 5. Conclusions

Our work demonstrates that chronic isoproterenol infusion induces a similar HF phenotype in young and elderly female mice, but elderly animals, unlike young, show impaired reverse remodeling upon beta-adrenergic withdrawal, with persistent cell hypertrophy, tissue fibrosis and cardiac dysfunction.

## Data Availability

Full data are available by requesting them authors at: begona.benito@vhir.org.
